# Growth and development of the mammary gland in mice—control of the insulin-like growth factor system by hormones and metalloproteases, and putative interference with micro RNAs

**DOI:** 10.1093/af/vfad024

**Published:** 2023-06-14

**Authors:** Andreas Hoeflich, Anne-Marie Galow, Julia Brenmoehl, Frieder Hadlich

**Affiliations:** Institute of Genome Biology, Research Institute for Farm Animal Biology (FBN), Wilhelm-Stahl Allee 2, 18196 Dummerstorf, Germany; Institute of Genome Biology, Research Institute for Farm Animal Biology (FBN), Wilhelm-Stahl Allee 2, 18196 Dummerstorf, Germany; Institute of Genome Biology, Research Institute for Farm Animal Biology (FBN), Wilhelm-Stahl Allee 2, 18196 Dummerstorf, Germany; Institute of Genome Biology, Research Institute for Farm Animal Biology (FBN), Wilhelm-Stahl Allee 2, 18196 Dummerstorf, Germany

**Keywords:** AKT, GH, metzincins, mTOR, prolactin

**Implications**

- Mouse models have improved our understanding of mammary gland growth and development under the control of endocrine signals.
- Micro RNAs (miRNAs) can influence the effects of hormone axes and protein families in the mammary gland.
- Due to the presence of miRNAs in exosomal vesicles, miRNAs can be exported from the mammary gland to peripheral and central tissues of the animal or to suckling offspring.
- It is necessary to identify the biological effects of milk miRNAs in the offspring but also in human consumers of dairy products, the latter can be considered a critical issue of food safety.

## Introduction

The insulin-like growth factor (IGF) system represents an important effector of mammary gland growth and development. Notably, in the mouse mammary gland, the IGF system integrates signals from growth hormone (GH) and prolactin. Within the cell, the IGF system impacts the pathway activating protein kinase B (AKT) and mammalian/mechanistic target of rapamycin (mTOR), which also mediates metabolic signals (Hou et al., 2021). Accordingly, mTOR holds a central position in controlling the growth, development, and metabolism of the mammary gland in mice. This most likely also applies to other mammal species since the pathway activating protein kinase B (AKT) and mammalian/mechanistic target of rapamycin (mTOR) is highly conserved among animals (Tatebe and Shiozaki, 2017). Our understanding of the control of IGF-related bioactivity has evolved significantly in the past. In addition to the peptide hormones (IGF1 and IGF2), IGF-receptors (IGF1R: IGF1 receptor and IGF2R: IGF2-mannose 6-phosphate receptor), six high-affinity IGF-binding proteins (IGFBP1 to 6), and the acid-labile subunit, we have to consider the effects of pregnancy association plasma proteins A and A2 (PAPPA and PAPPA2) and two stanniocalcins (STC1 and STC2), which block the IGFBP-proteolytic activity of PAPPAs (Argente et al., 2017). Moreover, IGF2 expression is controlled on the level of mRNA expression by IGF2 mRNA binding proteins (IGF2BP1 to 3). Finally, klotho and a novel IGFBP-like protein (IGFBPL1) seem to impact IGF-related activation of the mTOR pathway to complete the expanded IGF system, with 20 members included here in this work. The present review summarizes the current knowledge of IGF-related control of mammary gland growth and development in mice. In the second part of the review, we discuss the effects of miRNAs putatively targeting members from the IGF system during the development of the mouse mammary gland. Based on a recent contribution in the field (Wang et al., 2022), we discuss the potential control of signals from GH, prolactin, and metzincin metalloproteases by miRNAs.

### Functions of the IGF system for mammary growth, development, and function

**Figure 1.**
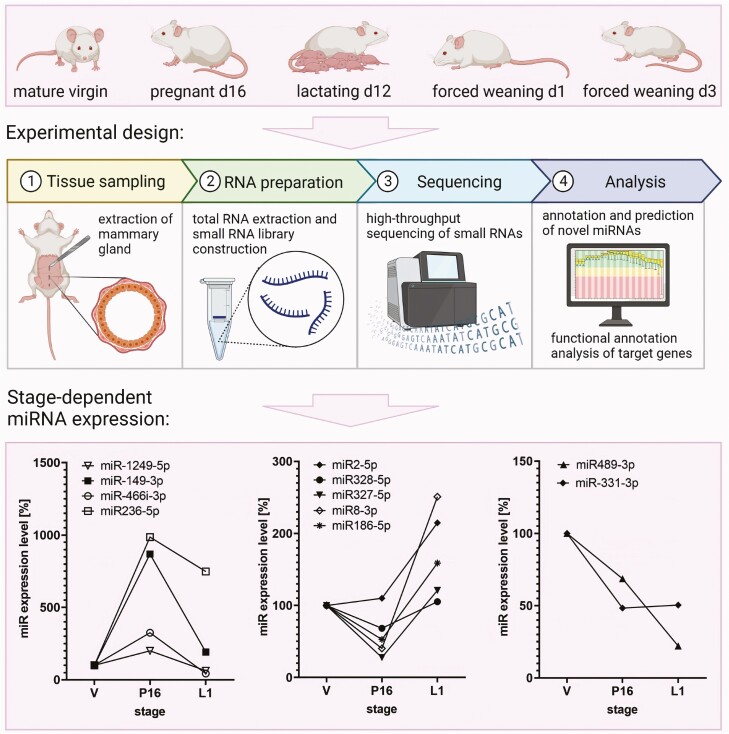
Design of the study published by Wang et al. (Wang et al., 2022). The mammary glands were studied at five defined stages of lactation from the virgin stage to weaning (d: day). RNA was extracted, and small RNA was sequenced and annotated to 852 known miRNA transcripts. In addition, 179 novel miRNAs were predicted. For all miRNAs, expression patterns were studied during mammary gland development. The lower panels provide examples for selected stages (V: virgin; P16: day 16 of pregnancy; L1: day 12 of lactation).

In the absence of the GH/IGF system, virtually no postnatal growth is observed in vertebrates. Accordingly, this system represents the most important endocrine system for controlling somatic growth. Under the control of GH and IGF, both cell size and cell number can be increased, thus defining the central parameters of tissue size. GH is produced in the pituitary gland and has direct effects on its target cells or tissues. In addition, GH can induce gene expression of IGF1 and thus impact growth by IGF-dependent mechanisms. Furthermore, tissue specificity is due to differential expression of IGFBPs, IGFBP-proteases, inhibitors of IGFBP-proteases, and calcium, as we rule out further down. In mice, IGFs, IGFBPs, and IGF1R are expressed in the virgin and the developing mammary gland, and cell-specific expression patterns have been described for IGFBPs (Wood et al., 2000). The permissive role of the IGF1R for normal development, duct branching, and alveologenesis of the mouse mammary gland has recently been reviewed in great detail (Bulatowicz and Wood, 2022). While IGF1 is strictly regulated by GH, IGF2 appears to mediate the effects of prolactin during mammary gland development and therefore is required for ductal branching and alveologenesis (Brisken et al., 2002; Hovey et al., 2003). Accordingly, the IGF2R may be perceived as an antagonist of prolactin actions since this receptor acts as a decoy receptor exclusively for IGF2. Also, the permissive function of particular IGFBP members for the growth and development of the mouse mammary gland has been provided. Thus, the lack of IGFBP3 in mice resulted in increased concentrations of phosphorylated AKT and increased tumor growth in the mammary gland (Blouin et al., 2015). IGFBP5 is also required for normal growth, especially for mammary gland regression in mice, as shown by local overexpression (Tonner et al., 2002), whereas the absence of IGFBP5 in knockout mice delayed apoptosis (Ning et al., 2007) and mammary gland remodeling. Moreover, proteolytic degradation of IGFBP5 by PAPPA has been linked to the delayed involution of the mammary gland in PAPPA transgenic mice (Takabatake et al., 2016). Overexpression of PAPPA in the mammary gland from transgenic mice was further associated with increased phosphorylation of AKT. Most interestingly, increased levels of phosphorylated AKT in PAPPA transgenic mice were not observed during lactation, which was attributed to elevated expression of STC1 and -2 (Takabatake et al., 2016). Accordingly, PAPPA, a member of the metzincin superfamily, defines the activities of the IGF system during mammary duct growth and gland involution in mice. The outstanding contribution by Takabatake et al. provided evidence for the role of the IGF system during mammary gland growth and development in mice but did not support a role for lipid and protein synthesis during lactation by the control of mTOR as discussed before (Hou et al., 2021). Nevertheless, earlier studies suggested the relevance of the IGF system for mammary blood flow, milk yield, and gastrointestinal development of neonates (Prosser et al., 1990; Prosser, 1996). The activity of the IGF system may further be regulated by klotho or IGFBPL1. In breast cancer cells, klotho has direct interactions with the IGF1R and thereby blocks IGF-related activation of AKT (Wolf et al., 2008). In addition, klotho acts as an inhibitor of calcium shuttling via store-operated channel entry (Shmulevich et al., 2020), which so far has not been considered with respect to the activity of PAPPAs. By contrast, IGFBPL1, which directly binds to IGF1, is required for calcium-dependent activation of mTOR, at least in retinal cultures from mice (Guo et al., 2018). Collectively, the expanded group of IGF superfamily members, including proteases, protease inhibitors, mRNA binding proteins, and proteins with an impact on the interaction of IGFs and the IGF1R, can be characterized at the level of signal transduction such as phosphorylation of the IGF1R. Thus, the overall IGF-related bioactivity may contain innovative biomarker information similar to free IGF (Frystyk, 2007; Jeyaratnaganthan et al., 2010). For the assessment of IGF-related activity, novel platforms are in service, which quantifies the activity of the complete system on the level of IGF1R activation (Chen et al., 2003). In addition, and directly related to the mTOR pathway, a highly sensitive bioassay was developed to measure AKT phosphorylation in biological body matrices, including milk (Walz et al., 2021). Recent work by Wang et al. (Wang et al., 2022) provided evidence for the regulation of the mTOR pathway on the level of miRNA during mouse mammary gland development from the virgin stage to lactation (Figure 1). In this study, complex control of the IGF system by miRNAs can be assumed in the developing mammary gland. In particular, out of 1031 regulated miRNAs, 172 miRNAs have putative interactions with one or more members of the expanded IGF system defined earlier in this paper. Thus, more than 16% of the regulated miRNome in the mammary gland appears to be related to the IGF system, and it is worthwhile to have a closer look at the identity and potential functions of these miRNAs and their putative or known targets.

### Identification of a miRNA signature potentially targeting the IGF system

The origins of life can be attributed to the inherent feature of RNA molecules to form comparably stable RNA/RNA structures (Bernhardt, 2012). Accordingly, miRNA binding to other RNA transcripts can be considered an ancient and fundamental concept to control biological processes or, more specifically, the activity of other RNA molecules. Since miRNAs can be shuttled to distant cells and tissues by means of exosomal vesicles, some authors attribute “hormone-like” functions to miRNAs. In the following section, we will discuss putative interactions of miRNAs with compounds from the IGF family, known as a “classical” hormonal system. In other words, we describe possible interactions between an ancient “hormone-like” system and a “classical” hormonal system, which expressed increasing complexity during the evolution of multicellular organisms.

**Figure 2.**
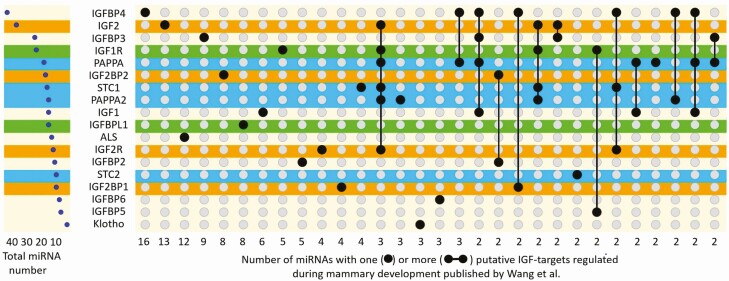
Hierarchy and interaction of IGF members as putative targets of miRNAs regulated during mouse mammary development, published by Wang et al. (2022). The left panel indicates the total number of miRNAs that putatively target individual IGF members. The right panel provides numbers of miRNAs commonly targeting either unique (isolated dots) or more than one IGF member (lines connecting different dots). Uncommon combinations (for single miRNAs) were not included in due to space limitations. Since IGFBP1 and IGF2BP3 represent putative targets of only one miRNA regulated or expressed during mammary development, both members are lacking in the presentation. Hierarchy and interaction extracted from supplementary table S4 published by Wang et al. were analyzed and visualized by the UpSetR plot (Conway et al., 2017). The putative miRNA targets were attributed particularly to GH (yellow), prolactin (orange), or metzincin effects (blue). Green color identifies putative miRNA targets that could be related both to GH and prolactin effects (IGF1R: IGF1 receptor; IGF2R: IGF2 receptor, IGFBP: IGF-binding protein; IGF2BP: IGF2 mRNA binding protein; IGFBPL: IGFBP-like; ALS: acid labile subunit; PAPPA/A2: pregnancy-associated plasma protein A/A2; STC: stanniocalcin; mTOR: mammalian/mechanistic target of rapamycin).

**Figure 3.**
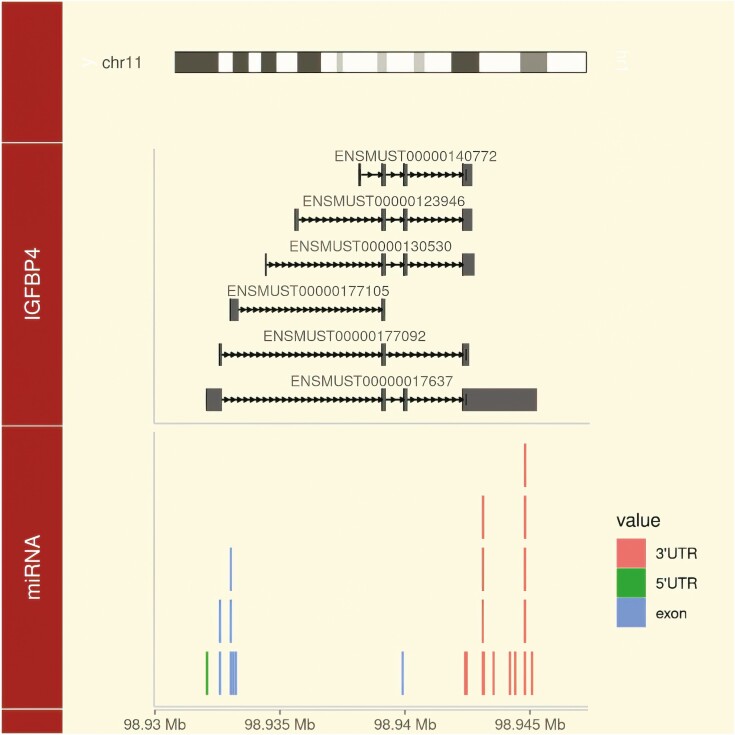
Predicted interactions of miRNAs expressed or regulated during mammary gland development in mice (Wang et al., 2022) with IGFBP4 RNA transcripts extracted from the Ensembl database (https://www.ensembl.org/) version 107. In the ggbio (Yin et al., 2012) schematic, only miRNAs with known miRBase (Kozomara et al., 2019) sequences were integrated. Their relative positions were predicted based on optimal sequence homology using RNAhybrid (https://bibiserv.cebitec.uni-bielefeld.de/rnahybrid/) version 2.1.2 within exonic regions. Relative positions on mouse chromosome 11 are provided at the bottom line. Untranslated regions are colored red or green; others are shown in blue (IGFBP4: insulin-like growth factor binding protein 4).

**Figure 4.**
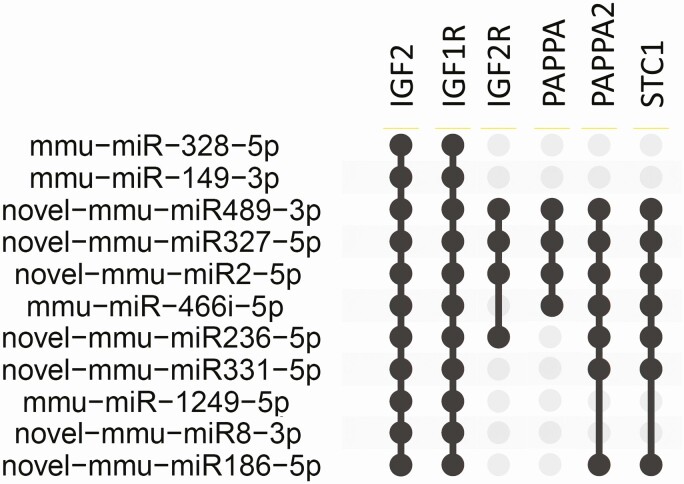
Eleven miRNAs regulated during mammary gland development in mice putatively target IGF1R and IGF2. A subset of miRNAs can further be related to IGF2R, PAPPA, and PAPPA2, as well as STC1. Data were extracted from supplementary table S4 published by Wang et al. (Wang et al., 2022) and analyzed and visualized by the UpSetR plot (Conway et al., 2017). Abbreviations are provided with the captions of Figure 2.

**Figure 5.**
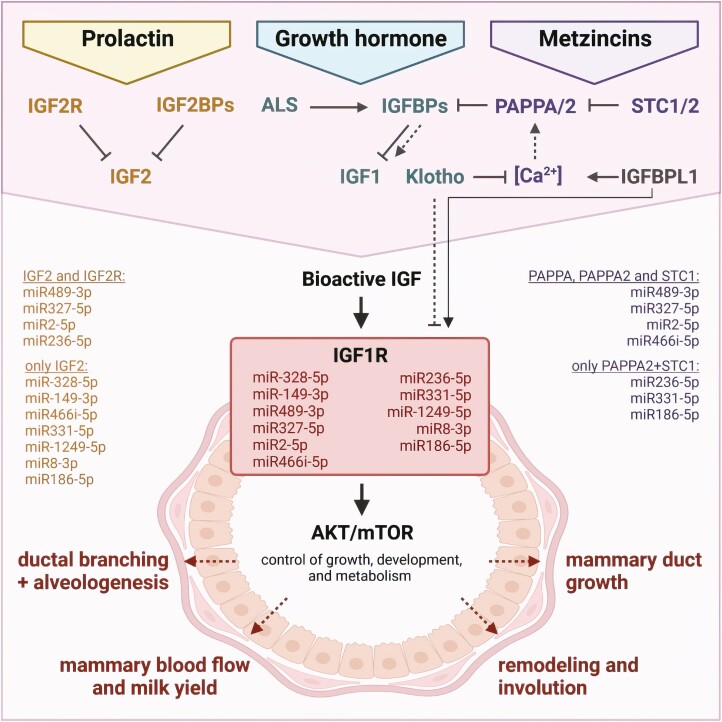
Multiple levels of mTOR control by miRNAs expressed or regulated during growth and development of the mouse mammary gland were published by Wang et al. (Wang et al., 2022). Signals from prolactin and GH can be integrated at the level of IGF-related bioactivity, which is further controlled by IGFBP-proteases from the metzincin family. Joint control by a subgroup of eleven miRNAs presented in Figure 4 is integrated into the schematic. Abbreviations are provided with the captions of Figure 2.

Intricate interactions of both systems during mammary gland development in mice can be deduced from the study of Wang et al. (Wang et al., 2022), demonstrating that each component of the expanded IGF system represents the putative target of at least one regulated miRNA. With respect to the IGF system, the greatest number of miRNAs identified by Wang et al. were related to IGFBP4 and IGF2 (Figure 2). IGFBP4 is a putative target of 42 different miRNAs, which also target 14 other IGF superfamily members, while IGF2 is a potential target of 36 miRNAs related to 15 other IGF superfamily members. This not only indicates the relevance of IGFBP4 and IGF2 in the mammary gland but also may indicate that prolactin signaling is regulated by miRNAs via the control of IGF2 mRNA during mouse mammary gland development as discussed earlier in this review (Brisken et al., 2002; Hovey et al., 2003). In the study published by Wang et al., exclusively interactions of miRNAs with the 3`untranslated region were considered. However, based on sequence homology and free energy calculated by *RNAhybrid* (https://bibiserv.cebitec.uni-bielefeld.de/rnahybrid/), interactions of miRNAs also with exonic mRNA can be postulated, which is provided as an example for different IGFBP4 RNA transcripts (Figure 3) extracted from the Ensembl database (https://www.ensembl.org/). Except for IGFBP1 and IGFBPL1, the members of the expanded IGF system are putative targets of several miRNAs, and several miRNAs regulate multiple members of the expanded IGF system. The greatest degree of interaction between miRNAs, published by Wang et al. (Wang et al., 2022), and their putative IGF targets were identified for a subset of 11 miRNAs and six members from the IGF system (Figure 4). Notably, from this subset of 11 miRNAs regulated or expressed during mammary gland development, all miRNAs can be linked to the expression of IGF2 and IGF1R. In addition, three novel miRNAs were identified by Wang et al., which further can be linked to the expression of IGF2R, PAPPA, PAPPA2, and STC1. Accordingly, we would like to propose a signature of miRNAs that coordinates prolactin, GH, and metzincin signaling on the level of the IGF system. By identifying IGF-related miRNAs expressed during mammary development in mice, we can collectively describe different levels of control by miRNAs (Figure 5).

### Local and distant effects of miRNAs expressed in the mammary gland

In contrast to classical hormones, the specificity of miRNAs is expressed on a much lower level since miRNAs can have thousands of putative gene targets (Lewis et al., 2005). The lack of specificity, however, seems to be connected with a great number of miRNAs genes, which in humans have been reported to be 550 (Fromm et al., 2022). Nevertheless, since multiple mature miRNA species can be processed from pre-miRNAs, the total number of mature miRNAs is reported by several sources to be in the thousands. Here, we review how miRNAs differentially expressed during mouse mammary gland growth and development might control IGF system activation, expression, and signaling. In addition, we discuss potential effects of miRNAs for local and distant control of growth and metabolism and thereby may get an impression of the fundamental control of biological processes by miRNAs.

In the signature of 11 miRNAs regulated during mammary development in mice (Wang et al., 2022), mmu-miR-328-5p has suggested interactions with additional six members from the superfamily of the IGF system (IGFBP3 and 4, IGF2BP1 to 3, and STC2). Thus, we may assume the effects of miR-328 not only on the level of IGF2 and IGF1R expression but also on IGFBP expression and stability, thereby regulating IGF-related bioactivity. In fact, an active role of miR-328 in the mammary gland may be presumed since miR-328 inhibited cell proliferation in human breast adenocarcinoma cells (cell line MDA-MB-231) (Luo et al., 2018). Moreover, miR328 was demonstrated to be involved in vascular development and remodeling in the rat lung (Guo et al., 2012). More specifically, miR-328 negatively affects lung smooth muscle cell proliferation, which is abrogated in response to hypoxic conditions (Xing et al., 2019). Interestingly, this is in line with one of the few postnatal functions described for IGF2 so far, namely the promotion of smooth muscle cell proliferation (Zaina et al., 2002). Since miR-328 has functions for both IGF1R and IGF2, this may point to a particular role of miR-328 for vascular growth and remodeling in specific tissues. Recently, calcium signaling has also been shown to be a regulator of the IGF system, as it is required for the activity of the metzincin superfamily member PAPPA-aa (Liu et al., 2020), which is essential for proteolytic IGFBP degradation and thus for the release of IGFs from their IGFBPs. It is thus interesting to note that miR-328 in smooth muscle cells reduced the activity of the voltage-dependent L-type calcium channel subunit alpha-1C (CaV1.2) (Guo et al., 2012), whereby miR-328 controls the activity of the IGF system. Another miRNA represented by a greater number of putative targets from the IGF superfamily (IGF1R, IGF2, IGFBP4 and 5, STC2, and IGFBPL1) is miR-149 (Wang et al., 2022). So far, biological functions of miR-149 were related to direct interactions with AKT1 (Zhang et al., 2014) or the Fas ligand (Tian and Yan, 2016), which resulted in tumor suppression in hepatocarcinoma patients or the induction of the apoptotic pathway in an acute myeloid leukemia cell line. In dairy cows, bta-miR-149-5p was part of a miRNA signature downregulated in the transition to lactation after calving and was discussed in the context of metabolic adaptation and mTOR signaling (Veshkini et al., 2022).

A direct link from miRNA to mTOR signaling has also been established for two additional members of the miRNA signature provided in Figure 4, namely for miR-1249 and miR-8. While miR-1249 is regulated by p53 and blocks colorectal cancer growth, invasion, and angiogenesis via AKT/mTOR signaling in mice (Chen et al., 2019), the link of miR-8 with AKT/mTOR signaling has only been demonstrated in Drosophila melanogaster (Hyun et al., 2009), where it is involved in the regulation of body size in response to steroids or metabolic hormones (Jin et al., 2012). Also, novel-mmu-miR-489-3p was identified in the signature of putative IGF-related effectors. In quiescent stem cells from the mammary gland of inbred mice, miR-489 is highly expressed and could be related to the maintenance of quiescence (Patel et al., 2019), as described in the muscle (Cheung et al., 2012). In fact, overexpression of miR-489 in the mouse mammary gland delayed growth of the ducts and formation of the end buds and, consequently, mammary gland development, with no effect on lactation (Patel et al., 2019). Remarkably, phosphorylation of AKT and mitogen-activated protein kinase (MAPK) was substantially suppressed in miR-489 transgenic mice. Therefore, it may be worthwhile to test the effects of this miRNA on the level of the IGF system in future studies considering the six putative mRNA targets. To date, for miR-2, miR-466i, or miR-331, effects on the level of the cell cycle, apoptosis, or malignant growth have been described in different cellular systems (Su et al., 2016; Jiang et al., 2021; Zhu et al., 2022). However, to the best of our knowledge, a clear connection with IGF effects or mammary development and function has not been provided so far. In breast cancer, miR-186 potently blocks the epithelial-mesenchymal transition (Sun et al., 2019), and in neuroblastoma cells, the IGF1 expression is reduced by miR-186 (Wang et al., 2018).

**Figure 6.**
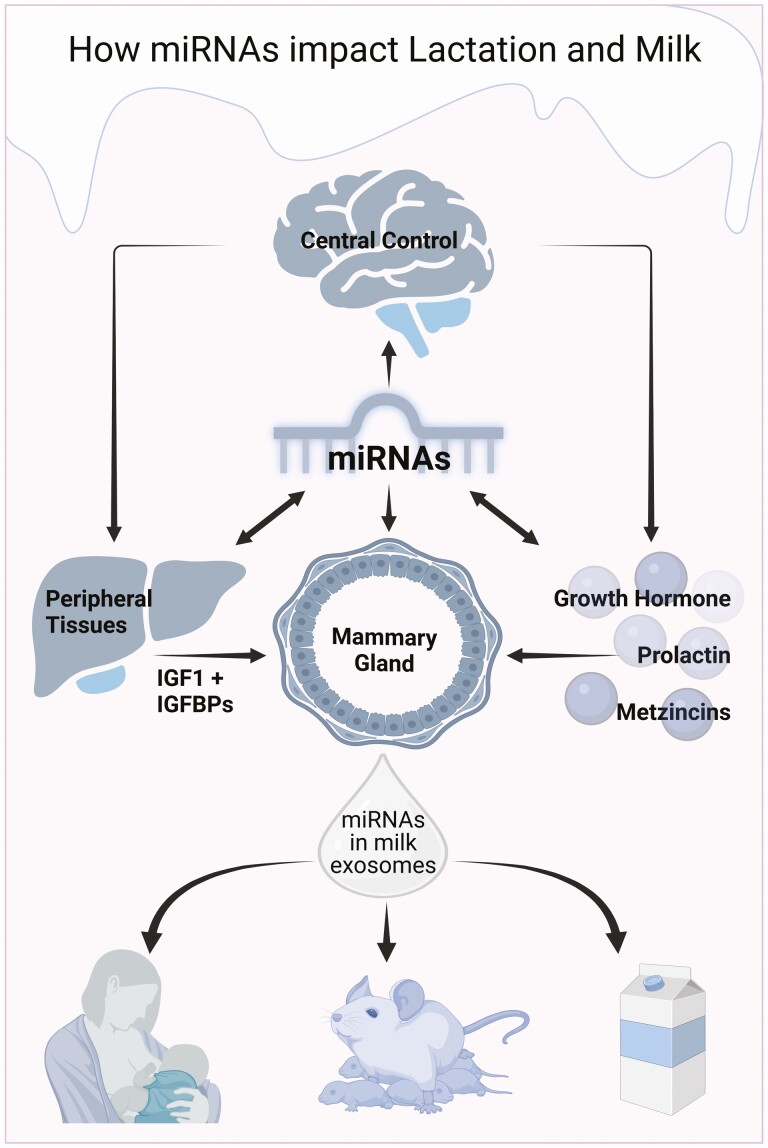
Interactions of central hormonal control with miRNAs. Neuroendocrine axes control the growth and development of the mammary gland by direct and indirect mechanisms in mice. At present, we are only beginning to realize the potential impact of miRNAs on endocrine pathways and their biological actions. This is an important issue because miRNAs originating from the mammary gland can access virtually any part of the body, including the brain. Through milk exosomes, miRNAs may also affect the infant or adult consumers of dairy products.

Finally, the effects of regulated miRNAs on the IGF system might not only affect the development and function of the mammary gland in female mice but also have implications for distant tissues (Figure 6). Indeed, exosomal vesicles derived from the mammary gland can be detected in the liver, lungs, spleen, and heart and even cross the blood-brain barrier in mice (Zhou et al., 2022). Accordingly, it is reasonable to consider both local and distant/systemic functions of miRNAs differentially expressed during mammary gland development. In addition, miRNAs can also be shuttled to the suckling or other consumers of mammary secretions. This may represent an important mechanism for controlling growth and development in the offspring. For example, intragastric administration of exosomes from porcine milk in mice induced gene expression of the IGF1R and intestinal development (Chen et al., 2016). Since for most of the miRNAs from the IGF-related signature, presented in Figure 4, the presence in exosomes has been published (Olave et al., 2016; Tian et al., 2021; Zhang et al., 2022), we have to consider their potential relevance also for the suckling. Eventually, exosomal miRNA species related to the mTOR pathway may also have side effects, e.g., in human milk consumers, as discussed by others in great depth (Melnik and Schmitz, 2019).

### Summary and outlook

A crucially missing piece of the puzzle of IGF-dependent control of mammary growth and development was provided by the integration of the metzincin family members PAPPA1 and 2 into the current concept of an expanded IGF system. It is thus possible to describe the interactions of IGFs and IGFBPs and longitudinal control of IGF-related activity by PAPPAs and STCs during growth and involution of the mammary gland in mice. Since metzincins can bind zinc and calcium (Bode et al., 1996), we need to integrate ion signaling into the future concept of the IGF system, and certainly not only in the mammary gland. It is becoming increasingly clear that the function of the IGF system cannot be adequately described by quantifying individual or selected IGF members. Instead, novel bioassays for the quantification of IGF-related systemic bioactivity have tremendous potential for the future. In particular, for the mammary gland, which is a comparatively recent achievement of vertebrate evolution, regulation of the IGF system via miRNome will also profoundly affect our understanding of mTOR control in the mammary gland. If we consider milk one of our most important nutrients, specific knowledge of biological functions is of critical importance. In the future, it may be interesting to study the expression of the miRNome at the cellular level by incorporating single-cell RNAseq. Another important task for the future may be to determine which miRNAs are present in extracellular vesicles and thus could have a local or systemic effect on the mother or developing infant, but possibly also on the consumer of dairy products.

## About the Authors

**Andreas Hoeflich** is Scientific Director at Research Institute for Farm Animal Biology (FBN) in Dummerstorf, Germany. Study of Biology at FAU in Erlangen-Nuremberg, followed by his PhD study at LMU Munich with the degree from TU Munich. Habilitation in Molecular Biotechnology (Gene Center, LMU Munich) and Animal Physiology (University of Rostock). Former Group Leader of the Mouse Genetics Group, and presently Head of the Signal Transduction Unit at FBN. He built up the new SPF-mouse facility at FBN, harboring transgenic and knockout models related to the GH/IGF-system. Member of the Growth Hormone and IGF-societies, International Buffalo Federation (IBF), and German Buffalo Federation (DBV). Andreas Hoeflich is dedicated to functional genome analysis with a particular focus on the GH/IGF-system in mammals.

**Anne-Marie Galow** is a Postdoc at Research Institute for Farm Animal Biology (FBN) in Dummerstorf, Germany. PhD in Cell Biology obtained at University Rostock after her diploma degree in Human Biology at University Greifswald. Formerly part of the DFG Research Training Group Welisa during PhD and postdoc in one of the EU-funded collaborative projects of the Excellence Research Program of Mecklenburg-Vorpommern, she is now member of the Signal Transduction Unit at FBN. Dr. Galow is engaged in the analysis of omics data to elucidate mechanisms of developmental processes and regeneration. For data processing, she relies on the latest tools developed within the scientific community and propagates sharing of data and code herself, generally advocating for the principles of Open Science.

**Julia Brenmoehl** is a scientist at the Research Institute for Farm Animal Biology (FBN) in Dummerstorf, Germany. She studied biotechnology at Ernst Abbe University in Jena, Germany, and biology at the University of Regensburg, Germany, followed by her PhD at the University Hospital Regensburg, Germany. Currently, she works with the worldwide unique long-term selected mouse lines established in Dummerstorf, Germany. Her research focuses on metabolism, more precisely energy metabolism, physical activity, lipid and glucose turnover, and mitochondrial biogenesis.

**Frieder Hadlich** is a research engineer at the Research Institute for Farm Animal Biology (FBN) in Dummerstorf, Germany. He studied Multimedia Technology at the Hochschule Wismar and Applied Computer Sciences at the University of Siegen. He has long experience as a bioinformatician, lecturer and research assistant at the University of Siegen, FZ Jülich and FBN Dummerstorf. Currently, Frieder Hadlich elaborates NGS-related pipeline solutions and enables efficient big data analysis in the context of functional genomics.
